# Bioactive Components from Qingwen Baidu Decoction against LPS-Induced Acute Lung Injury in Rats

**DOI:** 10.3390/molecules22050692

**Published:** 2017-04-26

**Authors:** Qi Zhang, Hai-Min Lei, Peng-Long Wang, Zhi-Qiang Ma, Yan Zhang, Jing-Jing Wu, Jing Nie, Su-Juan Chen, Wen-Jie Han, Qing Wang, Dan-Yang Chen, Cheng-Ke Cai, Qiang Li

**Affiliations:** 1 School of Chinese Materia Medica, Beijing University of Chinese Medicine, Beijing 100102, China; zhangqi060235@126.com (Q.Z.); hm_lei@126.com (H.-M.L.); wpl581@126.com (P.-L.W.); mazq1968@sina.com (Z.-Q.M.); m15600792986@163.com (J.N.); 18101257025@163.com (S.-J.C.); 17801080690@163.com (W.-J.H.); 17801085658@163.com (Q.W.); M17801080685@163.com (D.-Y.C.); 2 Fuzhou General Hospital of Chinese People ‘s Liberation Army, Fuzhou 350000, China; 125677870@163.com; 3 No. 476 Hospital of Chinese People ‘s Liberation Army, Fuzhou 350000, China; wcwjj@163.com

**Keywords:** Qingwen Baidu Decoction, acute lung injury, lipopolysaccharide, Principal Component Analysis

## Abstract

Qingwen Baidu Decoction (QBD) is an extraordinarily “cold” formula. It was traditionally used to cure epidemic hemorrhagic fever, intestinal typhoid fever, influenza, sepsis and so on. The purpose of this study was to discover relationships between the change of the constituents in different extracts of QBD and the pharmacological effect in a rat model of acute lung injury (ALI) induced by lipopolysaccharide (LPS). The study aimed to discover the changes in constituents of different QBD extracts and the pharmacological effects on acute lung injury (ALI) induced by LPS. The results demonstrated that high dose and middle dose of QBD had significantly potent anti-inflammatory effects and reduced pulmonary edema caused by ALI in rats (*p* < 0.05). To explore the underlying constituents of QBD, we assessed its influence of six different QBD extracts on ALI and analyzed the different constituents in the corresponding HPLC chromatograms by a Principal Component Analysis (PCA) method. The results showed that the pharmacological effect of QBD was related to the polarity of its extracts, and the medium polarity extracts E2 and E5 in particular displayed much better protective effects against ALI than other groups. Moreover, HPLC-DAD-ESI-MS*^n^* and PCA analysis showed that verbascoside and angoroside C played a key role in reducing pulmonary edema. In addition, the current study revealed that ethyl gallate, pentagalloylglucose, galloyl paeoniflorin, mudanpioside C and harpagoside can treat ALI mainly by reducing the total cells and infiltration of activated polymorphonuclear leukocytes (PMNs).

## 1. Introduction

Acute lung injury (ALI) and its severe form, acute respiratory distress syndrome (ARDS), are clinical syndromes characterized by acute-onset bilateral pulmonary infiltrates and non-cardiogenic lung edema [1], which cause the high mortality in critically ill patients [2]. With the high in-hospital morbidity and mortality, ALI represents a serious problem among patients admitted to intensive care units (ICUs) [3]. ALI is characterized by a sustained and uncontrolled inflammatory process in the lungs, thereby resulting in increased pulmonary vascular permeability, pulmonary edema and PMN infiltration into the alveolar space, which in turn impairs respiratory function [4]. A variety of clinical disorders, such as pneumonia, aspiration of gastric content, sepsis, major trauma and acute pancreatitis, can induce the occurrence of ALI [5]. The release of LPS from the outermost membrane of Gram-negative bacteria has been recognized as a principal pathogen in the pulmonary inflammation and sepsis process leading to ALI/ARDS [6,7]. Acute exposure to LPS provokes the innate immune system, leading to the activation of monocytes, neutrophils, and lymphocytes [8,9]. Then, the activation initiated a cascade of inflammatory cell influx, increased of cytokine release and lung capillary permeability, finally resulted in pulmonary edema [10,11]. So far, there is no specific therapeutic strategy for ALI, therefore, novel effective therapeutic agents are urgently required. In response, we have screened for potential drugs from among traditional Chinese medicines (TCMs) which have multi-target and multi-component characteristics for treating a disease. We found that Qingwen Baidu Decoction had the desired effect on the treatment of H1N1 influenza with satisfactory outcomes in the treatment of respiratory distress syndrome, and patient prognosis [12].

Qingwen Baidu Decoction (QBD), widely used as a treatment for acute lung injury in the Chinese community, consists of Gypsum Fibrosum, Cornu Bubali, Radix Rehmanniae, Rhizoma Coptidis, Fructus Gardeniae, Radix Platycodonis, Radix Scutellariae, Rhizoma Anemarrhenae, Radix Paeoniae Rubra, Radix Scrophulariae, Fructus Forsythia, Herba Lophatheri, Cortex Moutan Radicis and Radix Glycyrrhizae. QBD is an extraordinarily “cold” formula suitable for treating summer-heat syndrome in epidemic febrile disease [13]. It was traditionally used to cure epidemic hemorrhagic fever, intestinal typhoid fever, infectious mononucleosis syndrome leptospirosis, infectious atypical pneumonia, influenza, sepsis and so on [14]. In vivo and in vitro studies on the anti-inflammatory effects of QBD had been carried out in a great variety of acute and chronic inflammatory diseases [15,16,17]. These studies have demonstrated experimentally that QBD is an effective anti-inflammatory agent. Moreover, some studies showed an anti-inflammatory effect of QBD on ALI induced by LPS in rats [18,19,20]. In this study, we aimed to assess the pharmacological activity of different extracts of QBD on LPS-induced ALI in rats and elucidate the effective constituents of QBD against LPS-induced ALI by PCA analysis, which was able to simulate well the complex nonlinear relationships between the various chemical components and the pharmacological activity of herbal medicines, indicating good application prospects for developing quality evaluation methods which can reflect the efficacy of TCMs [21]. Firstly, we designed three doses of QBD for LPS-induced ALI in rats and selected a suitable dose of QBD. Secondly, we prepared different QBD extracts for treating LPS-induced ALI in rats and built HPLC chromatograms of six QBD extracts. Finally, we assessed the relationship between the pharmacological activity of the different QBD extracts and the effective constituents of QBD by PCA analysis.

## 2. Results

### 2.1. QBD Decreases LPS-Induced ALI in Rat

#### 2.1.1. QBD Decreases LPS-Induced Cell Infiltration

**Figure 1:**
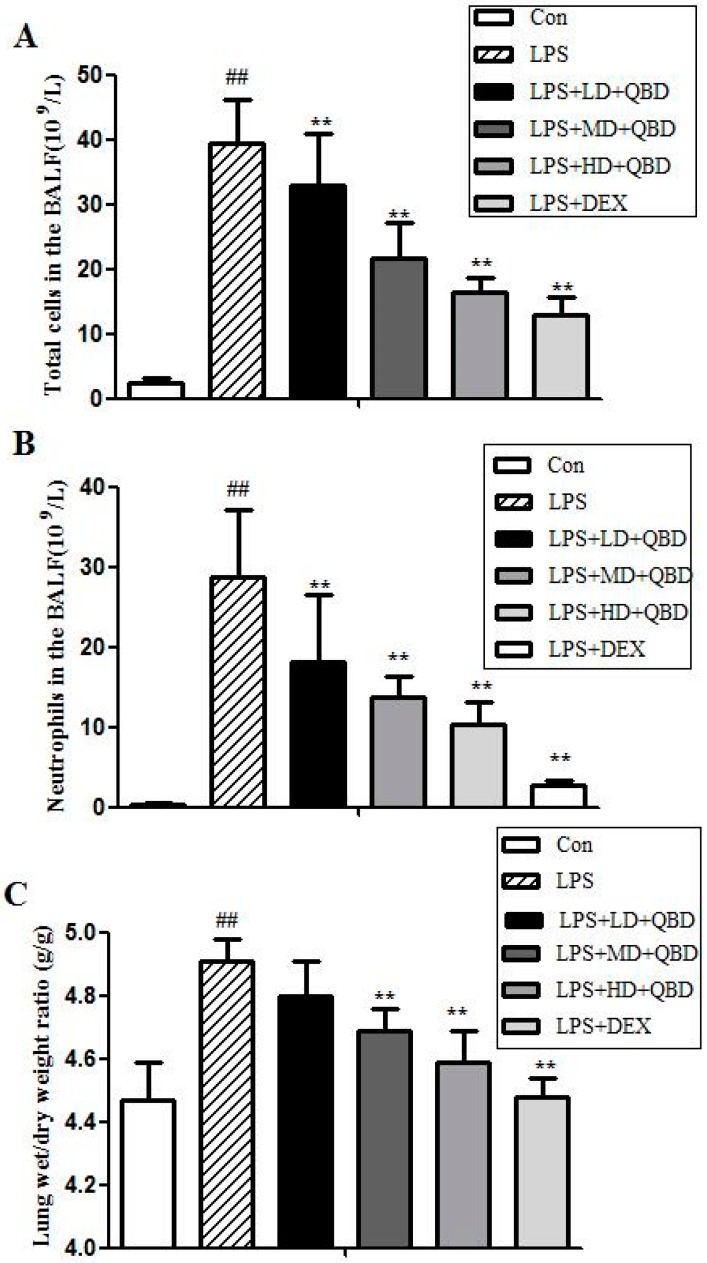

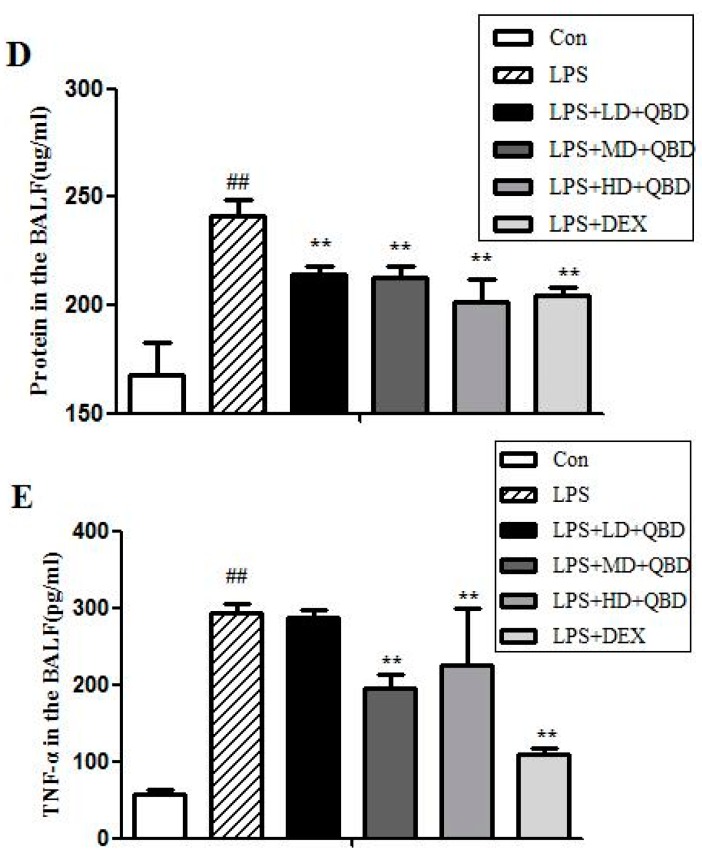
Effects of QBD pretreatment on LPS-induced ALI of the concentrations of inflammatory cytokines and edema in rat. Rats were given by gavage with DEX and different doses of QBD: LD (9.5 g), MD (19 g) and HD (38 g/kg) 24 h and 1 h before intratracheal instillation of LPS (5 mg/kg). Rats were anesthetized at 12 h after LPS challenge and BALF samples were obtained to analyze the inflammatory cytokines. The total leukocyte counts were greater in the LPS group than the control, LD, MD, HD, DEX group (**A**); the PMN counts in the LPS group were higher than the control, LD, MD, HD, DEX group (**B**); the wet/dry weight ratio in the LPS group was greater than the control, MD, HD, DEX group (**C**); the protein concentration was higher in the LPS group than the control, LD, MD, HD, DEX group (**D**) and the TNF-α level was greater in the LPS group than the control, MD, HD, DEX group. Data were presented as means ± SEM (*n* = 6). ## *p* ˂ 0.01 compared to control group; ** *p* ˂ 0.01 compared to LPS group (**E**).

Recruitment of neutrophils into the pulmonary compartment is an important feature of acute lung injury. As shown in Figure 1A,B, intranasal administration of saline was associated with few cell numbers in Bronchoalveolar Lavage Fluid (BALF), while LPS challenge caused a brisk and strong influx of neutrophils and total cells into BALF. Treatment of QBD (9.5, 19 and 38 g/kg) all significantly inhibited LPS-induced cell infiltration when compared with the LPS group (*p* < 0.05).

#### 2.1.2. QBD Attenuated LPS-Induced Protein Leakage and Pulmonary Edema

The wet-to-dry lung weight ratio (W/D ratio) is a parameter of pulmonary edema. As illustrated in Figure 1C, the lung wet/dry ratios were evidently higher after LPS challenge when compared with the control group (*p* < 0.05). However, pretreatment with QBD significantly reduced lung edema at the doses of 19 and 38 g/kg when compared with the LPS group (*p* < 0.05). Although 9.5 g/kg QBD also produced a reduction of lung edema as compared with the LPS group, it was not statistically significant. In addition, the inhibition of QBD (19 and 38 g/kg) was comparable to that produced by dexamethasone (DEX) treatment at 5 mg/kg (versus DEX group, *p* > 0.05).

As shown in Figure 1D, a marked increase of protein concentration in BALF was observed at 12 h after LPS instillation. However, in the three doses of QBD (9.5, 19 and 38 g/kg) treated groups, the increased protein level was partly inhibited as compared with the LPS group (*p* < 0.05).

#### 2.1.3. Effects of QBD on LPS-Induced TNF-α in BALF

As shown in Figure 1E, the levels of tumor necrosis factor-a (TNF-α) were evidently higher after the LPS challenge when compared with the control group. Although QBD (9.5 g/kg) produced a weak reduction as compared with LPS group, the median and high doses (19 and 38 g/kg) and DEX (5 mg/kg) significantly inhibited the increase of TNF-α levels in BALF compared with LPS group (*p* < 0.05).

#### 2.1.4. QBD Improved Lung Histopathology in ALI

**Figure 2:**
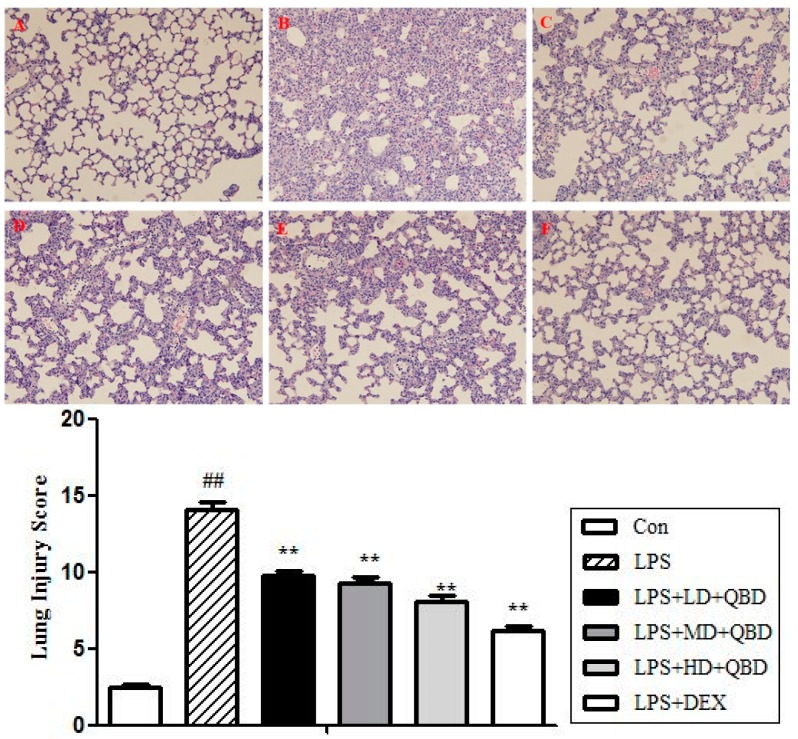
QBD ameliorated LPS-induced histological changes in the lung. Representative lung sections 12 h after LPS challenge were fixed, embedded in paraffin and cut into 5 μm slices. After H&E staining, histological examination was performed by light microscopy (**A**–**F**) at 100× magnification. It showed that QBD had obvious dose-effect relationship in improving lung histopathology in ALI: vehicle-treated rat (**A**); LPS group (**B**); LPS+DEX group (**C**); LPS + LD + QBD group (**D**); LPS + MD + QBD group (**E**) and LPS + HD + QBD group (**F**). ## *p* ˂ 0.01 compared to control group; ** *p* ˂ 0.01 compared to LPS group.

As shown in Figure 2, no evident histological alteration was observed in lung specimens of normal rat (A). However, the instillation of LPS resulted in significant lung injury, evidenced by the presence of alveolar hemorrhage, a marked swelling of the alveolar walls and remarkable recruitment of neutrophils into the alveolar spaces (B). The pathological changes were improved by DEX (C) and QBD administration (D–F). Meanwhile, it showed that QBD had obvious dose-effect relationship.

We had calculated Lung Injury Score (LIS) for the histology of QBD protecting ALI (Figure 2). The total surface of the slide was scored by two blinded pathologists with expertise in lung pathology in the following four categories: alveolar congestion, hemorrhage, neutrophil infiltration into the airspace or vessel wall, and thickness of alveolar wall/hyaline membrane formation. Each category was graded on a 0–4 scale, where 0 = no injury; 1 = injury up to 25% of the field; 2 = injury up to 50% of the field; 3 = injury up to 75% of the field; and 4 = diffuse injury [22]. A marked increase of lung injury score was observed at 12 h after LPS instillation (*p* < 0.01). However, three doses of QBD (9.5, 19 and 38 g/kg) treated group, it showed that alveolar congestion, hemorrhage, neutrophil infiltration into the airspace or vessel wall, and thickness of alveolar wall/hyaline membrane formation were improved as compared with the LPS group (*p* < 0.01).

### 2.2. Extracts of QBD Attenuated LPS-Induced ALI

#### 2.2.1. Extracts of QBD Decreased LPS-Induced Cell Infiltration

**Figure 3:**
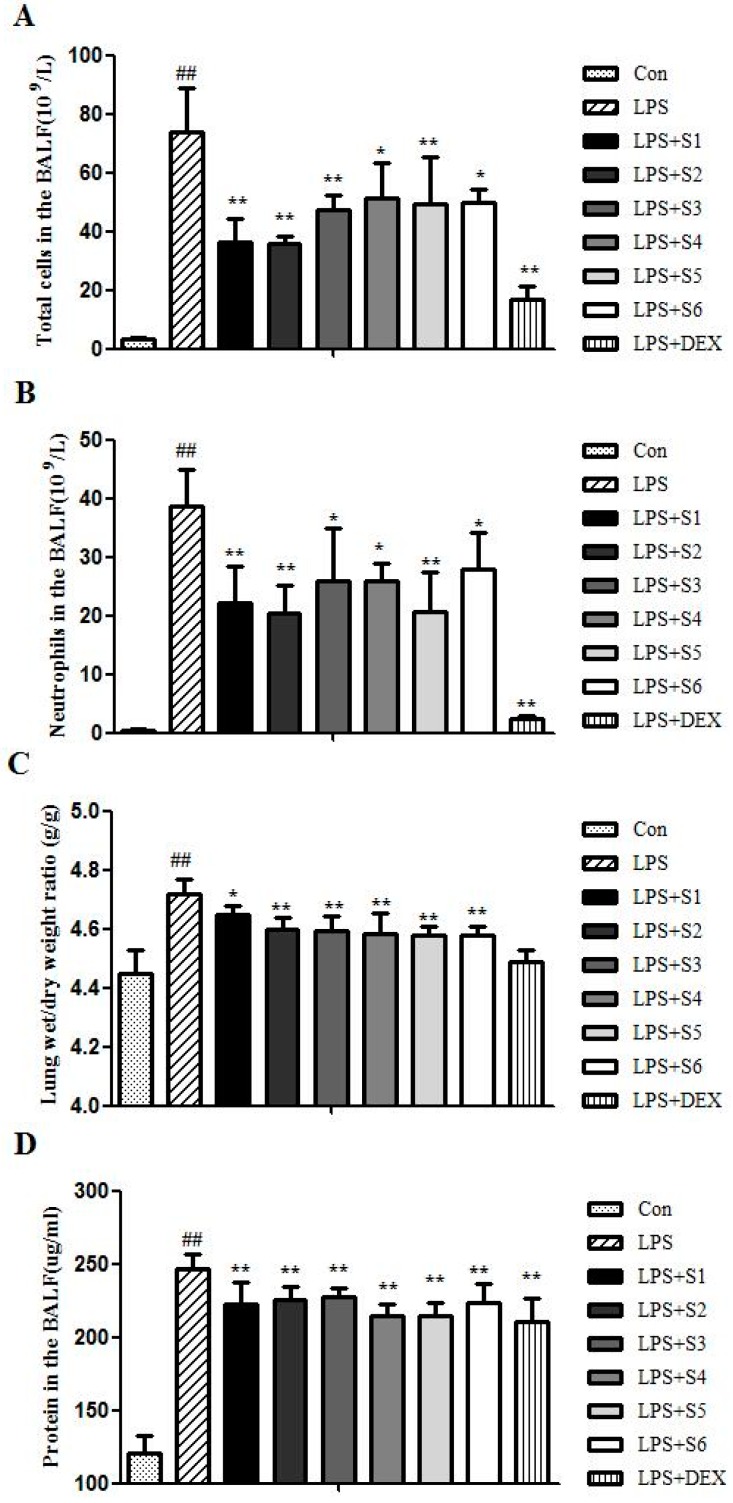
Effects of QBD on LPS induced lung injury and edema. Rats were administrated with different extracts of QBD (S1, S2, S3, S4, S5 and S6) 1h before intratracheal instillation of LPS (5 mg/kg). The total leukocyte counts were higher in the LPS group than the control, S1, S2, S3, S4, S5, S6, DEX group (**A**); PMN counts were higher in the LPS group than the control, S1, S2, S3, S4, S5, S6, DEX group (**B**); the wet/dry weight ratio was higher in the LPS group than the control, S1, S2, S3, S4, S5, S6, DEX group (**C**); and the protein concentration in BALF was greater in the LPS group than the control, S1, S2, S3, S4, S5, S6, DEX group (**D**). Data was presented as means ± SEM (*n* = 6). ## *p* ˂ 0.01 compared to control group; * *p* ˂ 0.05, ** *p* ˂ 0.01 compared to LPS group.

Recruitment of neutrophils into the pulmonary compartment is an important feature of ALI. As shown in Figure 3A,B, intranasal administration of saline was associated with few cell numbers in BALF, while LPS challenge caused a brisk and strong influx of total cells and neutrophils into BALF. Treatment of six extracts of QBD all significantly inhibited LPS-induced cell infiltration (*p* ˂ 0.01), especially S2.

#### 2.2.2. Extracts of QBD Attenuated LPS-Induced Protein Leakage and Pulmonary Edema

As shown in Figure 3C, a marked increase of protein concentration in BALF was observed after LPS instillation.

The six extracts of the QBD treated group all inhibited the increased protein level as compared with LPS group (*p* ˂ 0.01), especially S4. The lung W/D ratio in LPS group increased significantly when compared with the normal saline group, whereas the increase was significantly attenuated in different extracts of QBD treated group (*p* ˂ 0.01), especially S4 (Figure 3D). Therefore, extracts of QBD could suppress LPS-induced lung edema.

#### 2.2.3. Extracts of QBD Improved Lung Histopathology in ALI

**Figure 4:**
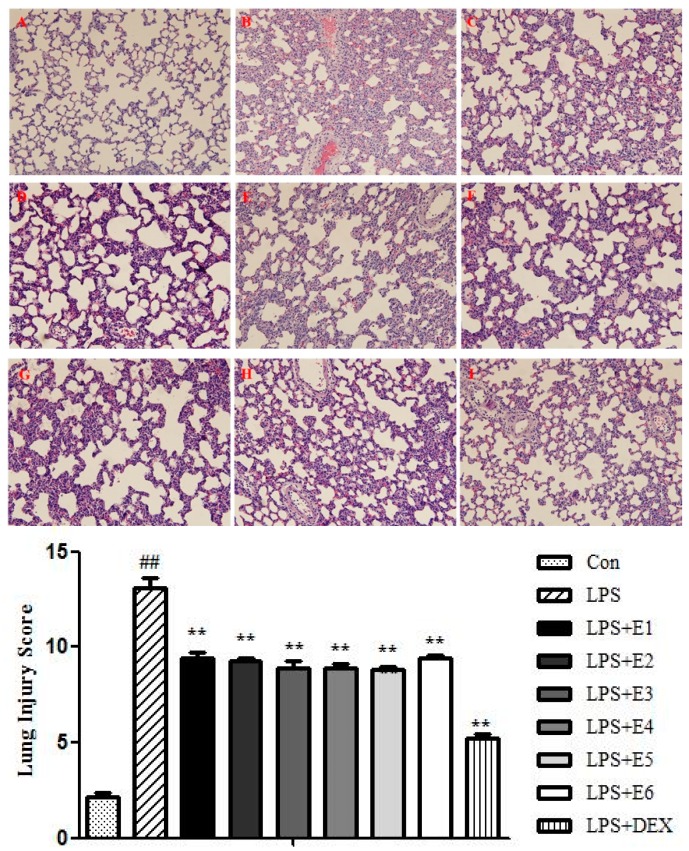
Extracts of QBD ameliorated LPS-induced histological changes in the lung. After H&E staining, histological examination was performed by light microscopy (**A**–**F**) magnification ×100. Lung sections were obtained from vehicle-treated rat (**A**); LPS group (**B**); LPS + DEX group (**C**); LPS + S1 group (**D**); LPS + S2 group (**E**); LPS + S3 group (**F**); LPS + S4 group (**G**); LPS + S5 group (**H**) and LPS + S6 group (**I**). ## *p* ˂ 0.01 compared to control group; ** *p* ˂ 0.01 compared to LPS group.

As shown in Figure 4, no evident histological alteration was observed in lung specimens of normal rat (A). However, the instillation of LPS resulted in significant lung injury, evidenced by the presence of alveolar hemorrhage, a marked swelling of the alveolar walls and remarkable recruitment of neutrophils into the alveolar spaces (B).

The pathological changes were improved by DEX (C) and six extracts of QBD administration (D–I). According to LIS (Figure 4), a marked increase of lung injury score was observed at 12 h after LPS instillation (*p* < 0.01). The six extracts of QBD treated group all improved the alveolar congestion, hemorrhage, neutrophil infiltration into the airspace or vessel wall, and thickness of alveolar wall/hyaline membrane formation as compared with the LPS group (*p* < 0.01).

### 2.3. Quality Control of Six Extracts in QBD

**Figure 5:**
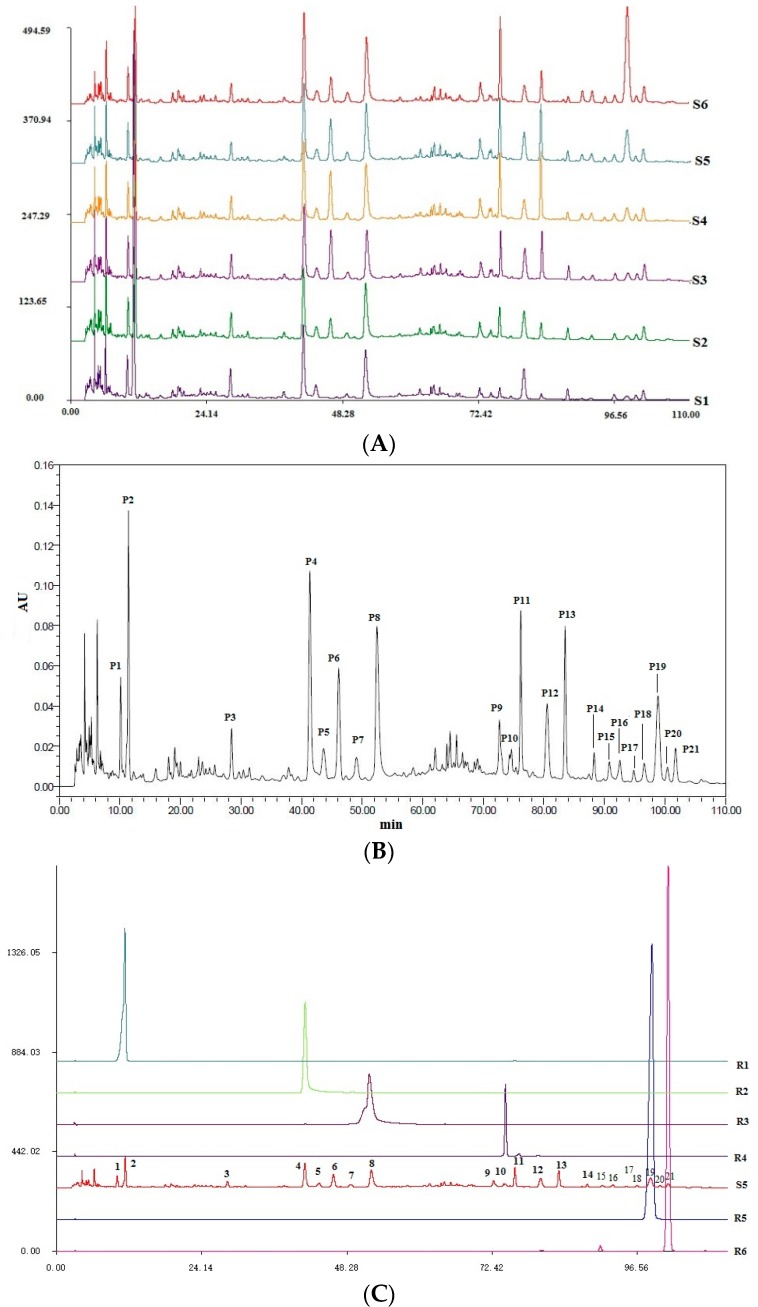
HPLC chromatograms. (**A**) HPLC chromatograms of six QBD extracts; (**B**) HPLC chromatogram of S5; (**C**) HPLC chromatograms of S5 and reference compounds. R1 (gallic acid); R2 (oxypaeoniflorin); R3 (paeoniflorin); R4 (verbascoside); R5 (paeonol); R6 (harpagoside).

HPLC chromatograms of six QBD extracts were obtained under u optimized conditions, and are shown in Figure 5A. Twenty one peaks with large areas and good resolution were collectively present in the six chromatograms and selected as the common characteristic peaks. According to the chromatograms, the 21 major peaks of all six QBD extracts were similar, but the peak areas were different and this discrepancy may lead to different degree of protection against ALI. In addition, they also had their own characteristic peaks, especially S1 and S6.

Twenty one peaks of S5 are shown in Figure 5B. Some compounds in the extracts were identified by comparison of their retention time and spectrum with the corresponding standard. By comparison with reference compounds, peaks 2, 4, 8, 10, 19 and 21 were identified as gallic acid, oxypaeoniflorin, paeoniflorin, verbascoside, paeonol and harpagoside, respectively (Figure 5C). They were also identified by HPLC-DAD-ESI-MS*^n^* (Table 1). Major constituents of active fractions were further isolated by preparative HPLC and purified. Some compounds, including pentagalloylglucose and ethyl gallate were identified by comparing their MS*^n^* and NMR data with previously reported data. NMR data of pentagalloylglucose and ethyl gallate are provided in the Supplementary Materials.

### 2.4. Chemical Structure Analysis by HPLC-DAD-ESI-MS^n^

**Figure 6:**
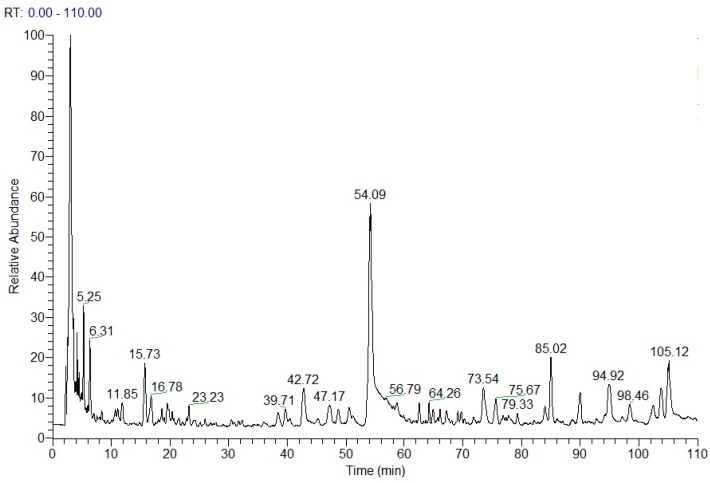
TIC of S5.

**Figure 7:**
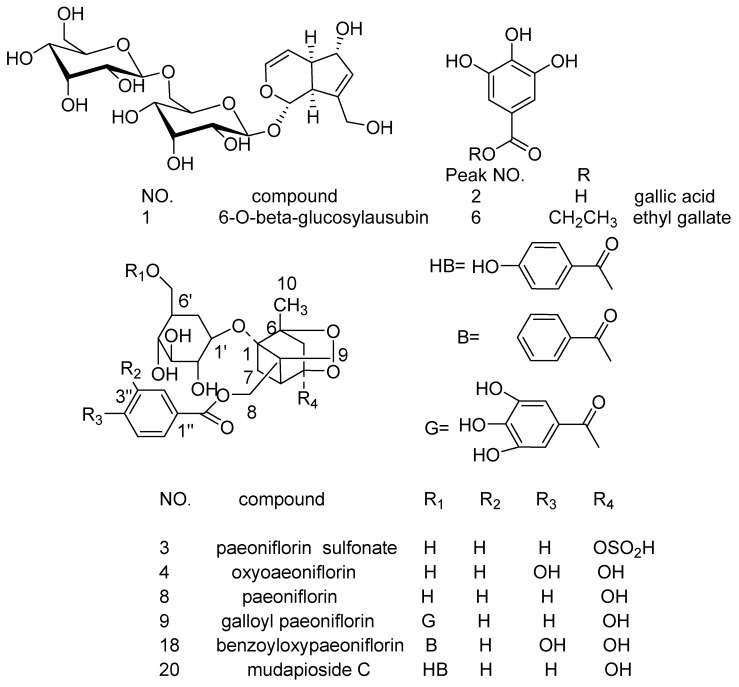

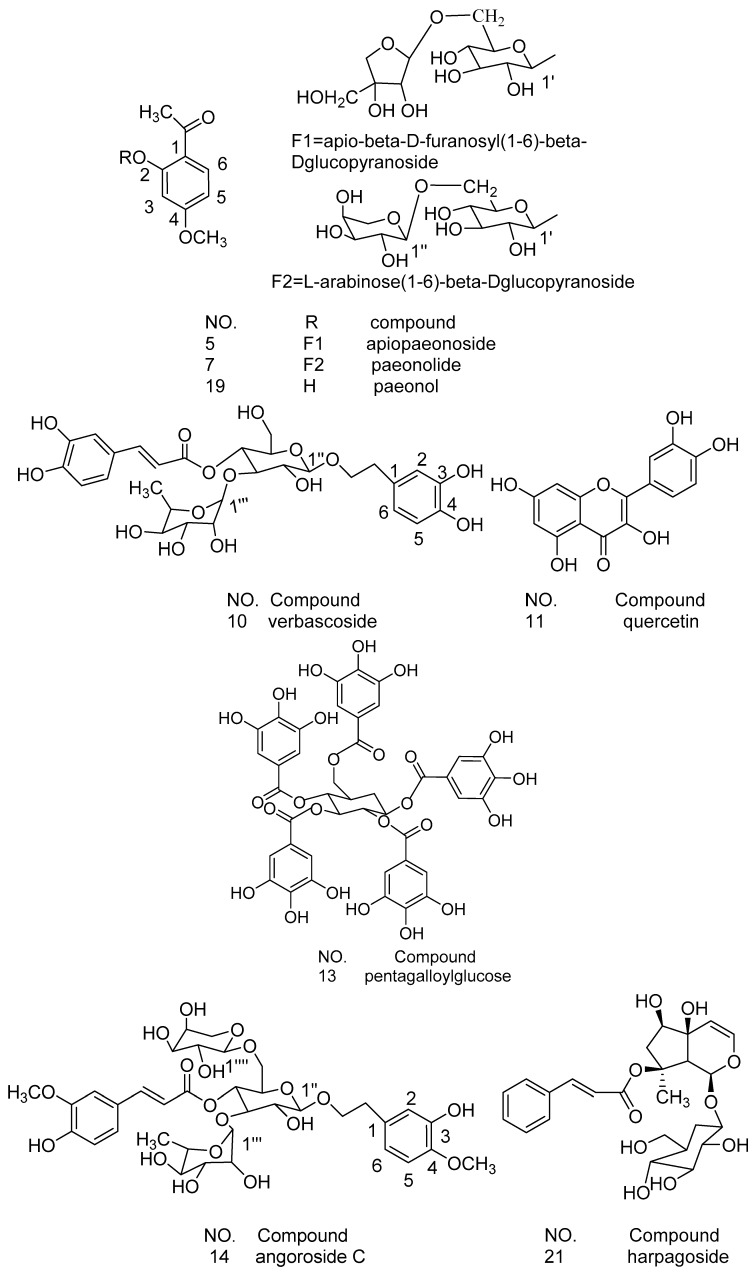
Structure of identified compounds.

Peak identification and assignment in HPLC chromatograms of QBD extracts were based on the comparison of their Rt and MS ion data with previously obtained data. The chemical structures of potential constituents were identified by HPLC-DAD-ESI-MS*^n^* (Tic in Figure 6) and listed in Table 1. The structure of identified compounds is shown in Figure 7.

### 2.5. The Analysis of Chemical Components Changes of Six Extracts in QBD from PCA

**Figure 8:**
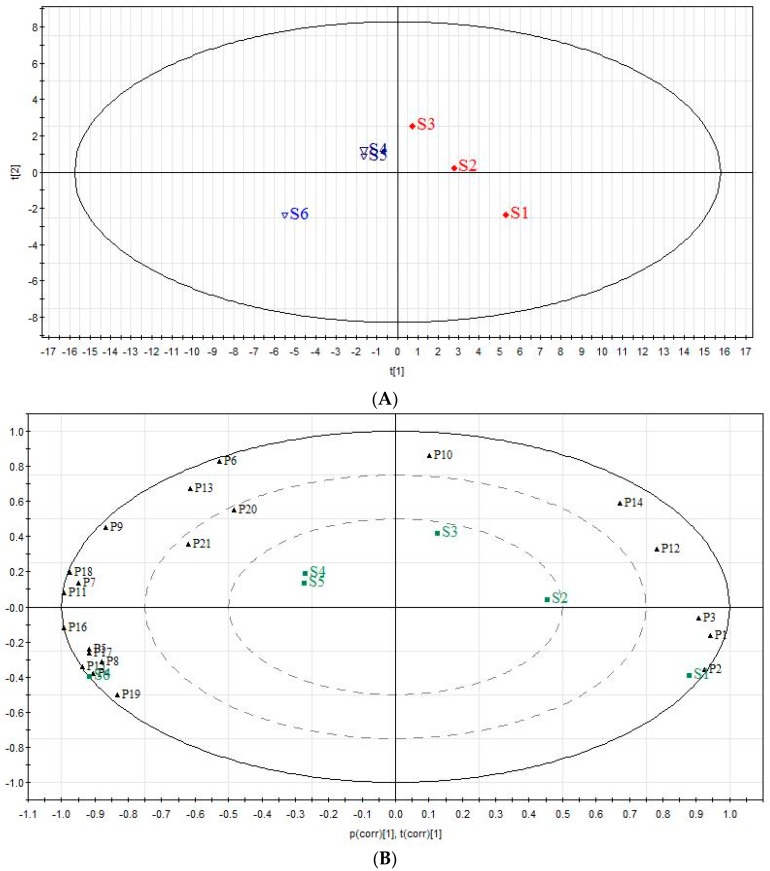
PCA score plot (**A**) and biplot (**B**) of six QBD extracts.

According to our work, there were no new components in the six extracts but the contents changed with different solvent extractions. In this paper, 21 chromatographic peaks were selected as characteristic components (*n* = 3), the relative peak areas (RPAs) of which were calculated for quantitative expression of the HPLC chromatograms. The HPLC chromatogram is shown in Figure 5A. PCA analysis on the RPA of 21 components was obtained for discrimination of the different extracts. For the UV data set of the first four principal components, the scores plot (Figure 8A) indicated that the samples could be classified into four clusters: S1, S2 and S3, S4 and S5, and S6, respectively. In the scores plot obtained by PCA, S6, S1, S2S3 and S4S5 were farther from each other. This is believed to be due to the fact that the content of the chemical constituents in S1–S6 were different.

To find the potential chemical markers for the discrimination among S1–S6, an extended statistical analysis was performed to generate the biplot (Figure 8B). The corresponding “biplot” analysis was used to visualize components which changed a lot [30]. Peaks 4, 5, 8, 15, 16, 17, 19, 7, 11 and 18, which showed increased intensities in S6, are the most important components to distinguish S6 from S1. Moreover, peaks 1, 2 and 3 were the most important components that differentiated S1 and S6. Meanwhile, the relative intensities of peaks 10, 12 and 14 were larger in S2 and S3. Meanwhile, peaks 6, 13, 9, 20 and 21 exhibited higher intensities in S4 and S5.

## 3. Discussion

LPS is known to induce the production of several inflammatory cytokines, tissue edema and injury [31]. Because LPS is the most important pathogen that leads to the development of ALI and ARDS, LPS aerosol inhalation was used in the present study to induce ALI in rats. Previous reports showed that LPS could cause marked pulmonary inflammation as an acute injury after 2 h [32]. Meanwhile, our modeling results also showed that the optimal time for building a rat ALI model was 12–24 h, so in this study, BALF and tissue samples were collected 12 h after LPS exposure.

In the present study, for the first time, the effect of QBD pre-treatment on LPS-induced ALI in rats was tested. The results demonstrate that three doses of QBD had potent anti-inflammatory effects in rats against ALI induced by LPS. To explore the underlying constituents of QBD responsible for the oberved beneficial effects, we assessed the influence of six different QBD extracts on rats with LPS-induced ALI and analyzed the different occurrence or concentrations of the constituents by a PCA method.

Firstly, the degree of pulmonary edema, which is evaluated as edema, is a typical symptom of ALI [6]. Our data showed that QBD extracts significantly decreased the lung W/D ratio and protein-rich exudation in BALF, which indicated that extracts of QBD could prevent the leakage of serous fluid into lung tissue and significantly reduce pulmonary permeability. Interestingly, the water-soluble extracts could more significantly decrease the lung W/D ratio and total protein content more than the other extracts, particularly the 50% alcoholic S4 extract. Secondly, the inflammatory cells were estimated in the BALF, which plays a central role in the pathogenesis of ALI [33]. As expected, rats induced by LPS presented a massive infiltration of inflammatory cells including neutrophils and lymphocytes in the lungs. Pre-treatment with QBD extracts significantly down-regulated total cells and neutrophils in the BALF. Fat-soluble extracts could more significantly reduce the total cells and the infiltration of activated PMNs, especially the 10% alcohol S2 extract. The results demonstrated that the activity of the extracts in QBD on ALI was correlated with the polarity of the extracts exemplified by the stronger activity of semi-polar extracts S2 and S5. Finally, this was supported by the histological analysis of the lungs. Data obtained from our study demonstrated the preventive effects of QBD against ALI induced by LPS.

In this paper, 21 chromatographic peaks were selected as characteristic peaks. In the scores plot obtained by PCA, the relative intensities of peaks 10, 12 and 14 was higher in S2 and S3. Verbascoside and angoroside C were earlier reported to exhibit in vitro and in vivo anti-inflammatory activity [34,35,36]. Meanwhile, peaks 6, 13, 9, 20 and 21 exhibited higher intensities in S4 and S5. Peaks 6, 13, 9, 20 and 21 were identified as ethyl gallate, pentagalloylglucose, galloyl paeoniflorin, mudanpioside C and harpagoside, which were able to treat ALI mainly by reducing the lung W/D ratio and total protein content. These polyphenols, including ethyl gallate, pentagalloylglucose and galloyl paeoniflorin, have been shown to significantly protect against ALI induced by LPS in vitro and in vivo [37,38,39]. Harpagoside also has anti-inflammatory activity [40].

## 4. Materials and Methods

### 4.1. Chemicals and Reagents

Lipopolysaccharide (*Escherichia coli*, serotype 055:B5) was acquired from Sigma-Aldrich (St. Louis, MO, USA). The ELISA kit for tumor necrosis factor-a (TNF-α) was acquired from Abcam (Cambridge, UK). Dexamethasone (DEX) was purchased from Tianjin KingYork Group Co. Ltd. (Tianjin, China). The Bio-Rad protein assay kit was from Bio-Rad Laboratories (Hemel Hempstead, UK). PBS and acetonitrile (HPLC grade) were purchased from Thermo Fisher Scientific (Waltham, MA, USA). Distilled water was purchased from Watson’s Food & Beverage Co. (Guangzhou, China). Formic acid, analytical grade, was obtained from Beijing Reagent Company (Beijing, China). Qingwen Baidu Decoction was purchased from Beijing Tong Ren Tang. (Beijing, China). Gallic acid, oxypaeoniflorin, paeoniflorin, verbascoside, paeonol and harpagoside were purchased from the National Institutes for Food and Drug Control (Beijing, China). SD male rats were purchased from Vital River Laboratories Co., Ltd. (Beijing, China). The LTQ-Orbitrap mass spectrometer was acquired from Thermo Fisher Scientific. NMR spectra were recorded on a Varian VNMRS 600 MHz (VARIAN, PaloAlto, CA, USA) in DMSO-*d*_6_ solvent using TMS as reference.

### 4.2. Animal Experiments

#### 4.2.1. Animals and Housing

SD male rats weighing 200 ± 20 g (age of 6 weeks) were separated into different groups by a randomized procedure and acclimatized for 1 week prior to treatment. The rats were maintained under standard laboratory conditions (temperature of 21–23 °C, relative humidity of 45–65%, and 12 h/12 h light/dark cycle) with food and water freely available. All animal experiments were performed according to the ethical guidelines suggested by the Institutional Animal Ethics Committee and Committee for the Purpose of Control and Supervision of Experiments on Animals, Ministry of Health, and Government of China.

#### 4.2.2. Preparation of QBD and Its Extracts

(1) Preparation of QBD

QBD is composed of *Gypsum Fibrosum* 30 g, *Cornu Bubali* 12 g, *Radix Rehmanniae* 12 g, *Rhizoma Coptidis* 4.5 g, *Fructus Gardeniae* 12 g, *Radix Platycodonis* 12 g, *Radix Scutellariae* 15 g, *Rhizoma Anemarrhenae* 15 g, *Radix Paeoniae* 15 g, *Radix Scrophulariae* 15 g, *Fructus Forsythia* 15 g, *Herba Lophatheri* 15 g, *Cortex Moutan Radicis* 12 g and *Radix Glycyrrhizae* 6 g. The crude drugs were identified and the voucher specimen (School of Chinese Materia, Beijing University of Chinese Medicine, Beijing, China) of the prescription for this study was an aliquot from the same batch. The crude components of the formula were extracted three times by refluxing with boiling distilled water (1:12, g/mL) for 1 h. The extract was filtered, concentrated, freeze-dried, and stored at 4 °C until use.

(2) Preparation of Different Extracts and Standard Solution

The ALI experiment indicated that QBD and the four recipes had the function of protecting ALI. The recipe was composed of *Cornu Bubali* 12 g, *Radix Rehmanniae* 12 g, *Radix Paeoniae* 15 g, *Radix Scrophulariae* 15 g, *Cortex Moutan Radicis* 12 g. The dried material of QBD was milled into fine powder. The powder (66 g) was macerated in boiling distilled water (S1), 10% ethanol (S2), 30% ethanol (S3), 50% ethanol (S4), 70% ethanol (S5) and 95% ethano l (S6) for 1 h (with occasional shaking), and then extracted with boiling ethanol of different concentrations three times (1 h). The solvent was mixed and then evaporated under reduced pressure to yield a residue. The extracts (0.66 g/mL) was stored at 4 °C until use. Each accurately weighed standard of six markers, gallic acid (R1), oxypaeoniflorin (R2), paeoniflorin (R3), acteoside (R4), paeonol (R5) and harpagoside (R6) were dissolved in methanol and diluted to a suitable concentration. The solutions were stored at 4 °C.

#### 4.2.3. Treatment of Animals

(1) QBD Decreased LPS-Induced ALI in Rat

The rats were divided randomly into six groups: (1) normal saline (NS) vehicle group (control group); (2) LPS challenged group (LPS group); (3) DEX (5 mg/kg, intraperitoneally, reference drug); (4) LD + QBD (9.5 g/kg), MD + QBD (19 g/kg), and HD + QBD (38 g/kg). QBD (9.5 g, 19 g or 38 g/kg in 0.1 mL distilled water/10 g body weight) was given intragastrically 48 h, 24 h and 1 h before the intraperitoneal injection of sterile saline (0.1 mL/10 g body weight) or LPS (5 mg/kg, in 0.1 mL NS/100 g body weight). Three different doses of the samples were determined on the basis of pilot studies. All rats were euthanized at 12 h after LPS or sterile saline instillation, and the samples were collected for subsequent analysis.

(2) Preparation of QBD Extracts

The rats were divided randomly into nine groups: (1) normal saline (NS) vehicle group (control group); (2) LPS challenged group (LPS group); (3) DEX (5 mg/kg, intraperitoneally, reference drug); (4) S1, S2, S3, S4, S5, and S6 + LPS. S1–S6 (6.6 g/kg in 0.1 mL distilled water/10 g body weight) was given intragastrically 48 h, 24 h and 1 h before the intraperitoneal injection of sterile saline (0.1 mL/10 g body weight) or LPS (5 mg/kg, in 0.1 mL NS/100 g body weight). The dose of the samples was determined on the basis of pilot studies. All rats were euthanized at 12 h after LPS or sterile saline instillation, and the samples were collected for subsequent analysis.

#### 4.2.4. Measurement of Cytokines, Cell Counts and Protein Concentration in BALF

At the end of the experiment, the right main bronchus was subsequently ligated and a catheter was inserted from the trachea into the left lung. Then, the left lungs were lavaged with 1 mL of autodaved PBS for five times .The recovery ratio of the fluid was about 80% (4 mL) and the BALF was immediately centrifuged at 300 g for 10 min at 4 °C and the supernatants were stored in −80 °C for analysis. The cell pellets were re-suspended in PBS, and the total cell number was counted using a standard hemocytometer. Meanwhile, the cell pellets were re-suspended in PBS, centrifuged onto slides, and stained for 10 min with Wright-Giemsa staining. The slides were quantified for macrophages, neutrophils, and lymphocytes by counting a total of 200 cells/slide at ×40 magnification as the differential cell count. The levels of TNF-α in BALF were determined by enzyme-linked immunasorbent (ELISA) kits. The protein concentration in BALF supernatants was measured by the Bradford method by using a Bio-Rad protein assay kit with bovine serum albumin (Sigma) as a standard.

#### 4.2.5. Wet-To-Dry Lung Weight Ratio (W/D Ratio)

After euthanasia of rat, the right upper lungs were immediately weighed to obtain the wet weight, and then placed in an oven at 60 °C for 48 h to obtain the dry weight. The ratio of the wet lung to the dry lung was calculated to assess lung edema.

#### 4.2.6. Histological Analysis of Lungs

At 12 h after instillation of LPS, the lung tissues were harvested and fixed with 10% buffered formalin, imbedded in paraffin, and sliced. After hematoxylin and eosin (H&E) stain, pathological changes of mammary tissues were observed under a light microscope.

### 4.3. Quality Control of Extracts

All samples were from the extracts in4.2.2. (1).Each dried sample was ground to a fine powder (40 mesh) using a pulverizer. A 0.66 g portion of each powdered sample was accurately weighed and fully dissolved with water and decanted into a 10 mL volumetric flask. The final solution was passed through a 0.22 μm PTFE filter prior to use. An aliquot of 20 μL of each sample solution and standard solution was injected into the HPLC system for analysis. The HPLC fingerprinting analysis was carried out on an Agilent Zorbax AQ-C18 column (250 mm × 4.6 mm, 5 μm). A binary gradient elution mobile phase system composed of acetonitrile as solvent A and 0.1% formic acid in water as solvent B. The HPLC chromatogram elution profile was as follows: 0–10 min, 2% A; 10–20 min, 2–7% A; 20–40 min, 7–9% A; 40–50 min, 9–10% A; 50–60 min, 10–16% A; 60–65 min, 16–17% A; 65–80 min, 17–20% A; 80–112 min, 20–24% A. The mobile phase flow rate was 1.0 mL/min, and the UV spectra were 254 nm. In order to obtain a large amount of detectable peaks on the HPLC chromatogram, the spectra of all peaks in the chromatogram of QBD were detected with a photodiode array. The result was shown in Figure 3, and 254 nm was selected as detection wavelength by comparing different wavelengths.

### 4.4. Dereplication by HPLC-DAD-ESI-MS^n^

Peak identification and assignment in HPLC chromatograms of QBD were performed on an LTQ-Orbitrap mass spectrometer connected to the HPLC instrument via an ESI interface. The ionization parameters were set as follows: negative ion mode; with the tune method set as follows: sheath gas (nitrogen) flow rate of 30 arb, aux gas (nitrogen) flow rate of 5 arb, spray voltage 4.0 kV, capillary temperature of 350 °C, capillary voltage 25 V, tube lensvoltage 110 V. The on-line mass analyzer scanned the mass range *m*/*z* 50–1000. The collision energy for collision induced dissociation (CID) was adjusted to 30% of maximum intensity peak, and the isolation width of precursor ions was at *m*/*z* 2.0 Da. The dynamic exclusion to prevent repetition was enabled, and the repeat count was set at 5 with the dynamic repeat time at 30 s and dynamic exclusion duration of 60 s.

### 4.5. Principal Component Analysis

To compare the differences among extracts of QBD, unsupervised principal component analysis was performed based on the relative peak areas in the HPLC fingerprints chromatography using the Umetrics software from MKS Data Analytics (C Malmö, Sweden). Chemical markers were detected by using the PCA biplots [29].

### 4.6. Statistical Analysis

The mass data analysis was processed using Thermo Xcalibur 2.1 workstation (Thermo Fisher Scientific) for peak detection and peak alignment. All data was expressed as mean ± standard deviations and analyzed with one-way analysis of variance (ANOVA) using the SPSS v. 17.0 software (IBM, Armonk, New York, NY, USA). *p* < 0.05 was considered statistically significant.

## 5. Conclusions

The study aimed to discover the differences in the constituents of different extracts of QBD and their pharmacological effect in an ALI rat model induced by LPS, particularly on anti-inflammatory activity and edema inhibition effect. It was important to note that this study had demonstrated that the pharmacological effect of QBD was related to the polarity of its extracts, especially the semi-polar extracts S2 and S5. Moreover, the result of PCA analysis showed that verbascoside and angoroside C played a key role in reducing pulmonary edema, while, ethyl gallate, pentagalloylglucose, galloyl paeoniflorin, mudanpioside C and harpagoside were able to treat ALI mainly by reducing the total cells and the infiltration of activated PMNs. The current study also provided evidence supporting the idea that the different extracts should be prescribed differently in the clinic.

## Figures and Tables

**Table 1:** Identification of potential constituents in Qingwen Baidu Decoction.

No	Rt (min)	Molecular Formula	Molecular Weight (*m*/*z*)	Observed Ion Peaks (*m*/*z*)	Identified Compound
P1	10.78	C_21_H_32_O_14_	508.1787	553.1783[M + HCOO]^−^, 507.1732[M-H]^−^	6-*O*-β-glucosyl-aucubin [23]
P2	11.85	C_7_H_6_O_5_	170.0210	169.0145[M-H]^−^, 124.8813[M-COO]^−^, 97.1030[M-COO-CO]^−^, 80.9518[M-COO-CO-HO]^−^	gallic acid [24,25]
P3	29.04	C_23_H_28_O_13_S	544.1245	543.1188[M-H]^−^, 421.1752[M-H-C_7_H_6_O]^−^	paeoniflorin sulfonate [24,25]
P4	42.72	C_23_H_28_O_12_	496.1575	495.1511[M-H]^−^, 465.1869[M-H-HCO]^−^, 333.1177[M-H-glc]^−^	oxypaeoniflorin [24,25,26]
P5	45.19	C_20_H_28_O_12_	460.1575	459.1588[M-H]^−^, 505.1576[M + HCOO]^−^	apiopaeonoside [24,27]
P6	47.17	C_9_H_10_O_5_	198.0523	197.0457[M-H]^−^, 168.9350[M-H-C_2_H_5_]^−^	ethyl gallate [23]
P7	50.52	C_20_H_28_O_12_	460.1575	459.1523[M-H]^−^, 505.1576[M + HCOO]^−^	paeonolide [24,27]
P8	54.09	C_23_H_28_O_11_	480.1626	525.1609[M + HCOO]^−^, 478.9111[M-H]^−^, 449.1719[M-H-CH_2_O]^−^	paeoniflorin [23]
P9	73.54	C_30_H_32_O_15_	632.1736	631.1671[M-H]^−^, 613.2833[M-H-H_2_O]^−^, 491.2346[M-H-H_2_O-C_7_H_6_O_2_]^−^	galloyl paeoniflorin [24,26,27]
P10	75.65	C_29_H_36_O_15_	624.2049	623.2987[M-H]^−^, 461.2122[M-H-caffeoyl]^−^, 315.1192[M-H-caffeoyl-rhamnosyl]^−^	verbascoside [23]
P11	77.38	C_15_H_10_O_7_	302.0421	300.9996[M-H]^−^, 228.9682[M-H-O-2CO]^−^, 256.9158[M-H-O-CO]^−^	quercetin [24,27]
P12	81.38	C_24_H_28_O_12_		507.1525[M-H]^-^	unknown
P13	85.02	C_41_H_32_O_26_	940.1176	939.1130[M-H]^−^, 769.1382[M-H-C_7_H_6_O_5_]^−^	pentagalloylglucose [25,27,28]
P14	89.98	C_36_H_48_O_19_	784.2784	783.2723[M-H]^−^, 607.3094[M-H-feruloyl]^−^, 589.3885[M-H-feruloyl-H_2_O]^−^, 461.1645, [ferulic acid–H]^−^	angoroside C [29]
P15	92.68	C_41_H_30_O_25_		923.1136[M + H]^+^	unknown
P16	94.92	C_47_H_66_O_3_		679.5094[M + H]^+^	unknown
P17	97.15	C_52_H_60_O_2_		717.4650[M + H]^+^	unknown
P18	98.46	C_30_H_32_O_13_	600.1837	599.1782[M-H]^−^, 447.2278[M-H-C_7_H_6_O_2_-HCHO]^−^, 477.1727[M-H-C_7_H_6_O_2_]^−^	benzoyloxy-paeoniflorin [24,28]
P19	99.88	C_9_H_10_O_3_	166.0624	167.0695[M + H]^+^, 148.8797 [M + H-H_2_O]^−^,	paeonol [24]
P20	102.44	C_30_H_32_O_13_	600.1837	599.1783[M-H]^−^, 477.1727[M-H-C_7_H_6_O_2_]^−^, 447.2278[M-H-C_7_H_6_O_2_-HCHO]^−^	mudanpioside C [24]
P21	103.77	C_24_H_30_O_11_	494.1783	493.1729[M-H]^−^, 345.1204[M-H-cinnamic acid]^−^	harpagoside [29]

## Supplementary Materials

Supplementary materials are available online.

## Abbreviations

The following abbreviations are used in this manuscript:

QBD	Qingwen Baidu Decoction
ALI	Acute Lung Injury
LPS	Lipopolysaccharide
PCA	Principal Component Analysis
PMNs	polymorphonuclear leukocytes
ARDS	Acute respiratory distress syndrome
ICU	intensive care units
TCMs	traditional Chinese medicines
BALF	Bronchoalveolar Lavage Fluid
W/D ratio	wet-to-dry lung weight ratio
TNF-α	tumor necrosis factor-a
DEX	Dexamethasone
RPA	relative peak areas
NS	normal saline
CID	collision induced dissociation
ANOVA	one-way analysis of variance
