# Supplementary material for: Bioactive Components from Qingwen Baidu Decoction against LPS-Induced Acute Lung Injury in Rats
Source: Molecules. 2017 Apr 26;22(5):692. doi: 10.3390/molecules22050692 (PMC6154387; doi:10.3390/molecules22050692)
Supplement: Supplementary file 1 [file molecules-22-00692-s001.pdf]

X13:

The ^{1}H and ^{13}C NMR assignments of X13 were determined on the basis of the ^{1}H, ^{13}C, ^{1}H–^{1}H COSY, HMQC, and HMBC spectra. The ^{1}H and ^{13}C NMR, and ESI-MS data of 1 were identical to those of 1, 2, 3, 4, 6-penta-O-galloyl-b-D-glucose ($C_{41}H_{32}O_{26}$) in the literature [1] as follows: ^{1}H NMR (DMSO, 600 MHz): d 4.46 (1H, dd, J = 10 Hz, glc. H-6a), 4.75 (1H, m, glc. H-5), 4.46 (1H, d, J = 10 Hz, glc. H-6b), 5.59 (1H, t, J = 9.0, glc. H-2), 5.61 (1H, t, J = 9.0, glc. H-4), 6.13 (1H, t, J = 9.0, glc. H-3), 6.54 (1H, d, J = 9.0, glc. H-1), 6.93, 6.98, 7.00, 7.07, 7.13 (each 2H, s, galloyl H); ^{13}C NMR (DMSO, 150 MHz): 91.73 (glc. C-1), 67.79 (glc. C-4), 70.6 (glc. C-2), 71.96 (glc. C-3), 72.16 (glc. C-5), 61.59 (glc. C-6), 108.83, 108.93, 108.78, 108.75, 109.06 (galloyl C-2, C-6), 118.93, 118.13, 118.1, 117.96, 117.38 (galloyl C-1), 138.81, 139.16, 138.95, 139.19, 139.7 (galloyl C-4), 145.6, 145.54, 145.42, 145.15, 145.70 (galloyl C-3, C-5), 165.5, 164.5, 164.9, 164.6, 163.97 (–COO–); ESI-MS m/z939.1130[M-H]$^-$,769.1382[M-H-$C_7H_6O_5$]$^-$.

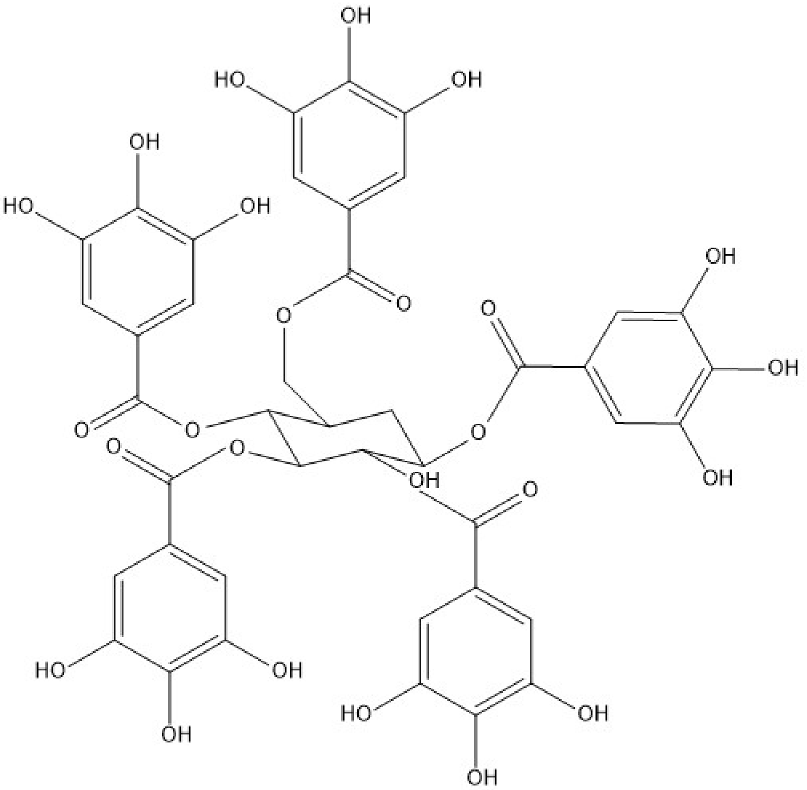

pentagalloylglucose

X6:

The ^{13}C NMR assignments of X6 were determined on the basis of the ^{13}C NMR, and ESI-MS data of X6 were identical to those of ethyl gallate ($C_9H_{10}O_5$) in the literature[2] as follows: ^{13}C NMR (DMSO,150MHz):119.9(C-1),108.3(C-2),145.63(C-3),138.58(C-4),145.63(C-5),108.27(C-6),168.04(C-7),60.64(C-8),14.17(C-9). ESI-MS m/z 197.0457[M-H]$^-$, 168.9350[M-H-C_2H_5]$^-$.

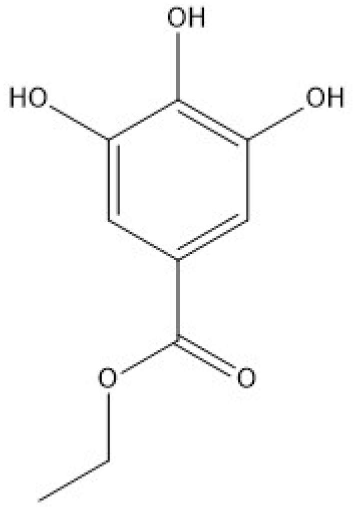

ethyl gallate

[1] J.Y. Cho, M.J. Sohn, J.K. Lee, W.G. Kim. Isolation and identification of pentagalloylglucose with broad-spectrum antibacterial activity from Rhus trichocarpa Miquel. Food Chemistry, 2010,123: 501–506.

[2] M. L. Ren.Study on chemical constituents and Bioactivities of Radix Paeoniae Alba. SHENYANG PHARMACEUTICAL UNIVERSITY, ShenYang,2009.
